# Stimulating the Melanocortin System in Uveitis and Diabetes Preserves the Structure and Anti-Inflammatory Activity of the Retina

**DOI:** 10.3390/ijms24086928

**Published:** 2023-04-08

**Authors:** Tat Fong Ng, Andrew W. Taylor

**Affiliations:** Department of Ophthalmology, Boston University Chobanian and Avedisian School of Medicine, Boston, MA 02118, USA

**Keywords:** melanocortins, uveitis, diabetic retinopathy, alpha-melanocyte stimulating hormone, alpha-melanocyte stimulating hormone-analog

## Abstract

The endogenous neuropeptide α-Melanocyte Stimulating Hormone (α-MSH) is a potent suppressor of inflammation and has an essential role in maintaining the normal anti-inflammatory microenvironment of the retina. While the therapeutic use of α-MSH peptide in uveitis and diabetic retinopathy models has been demonstrated, its short half-life and instability limit its use as a therapeutic drug. A comparable analog, PL-8331, which has a stronger affinity to melanocortin receptors, longer half-life, and, so far, is functionally identical to α-MSH, has the potential to deliver melanocortin-based therapy. We examined the effects of PL-8331 on two mouse models of retinal disease, Experimental Autoimmune Uveoretinitis (EAU) and Diabetic Retinopathy (DR). PL-8331 therapy applied to mice with EAU suppressed EAU and preserved retinal structures. In diabetic mice, PL-8331 enhanced the survival of retinal cells and suppressed VEGF production in the retina. In addition, retinal pigment epithelial cells (RPE) from PL-8331-treated diabetic mice retained normal anti-inflammatory activity. The results demonstrated that the pan-melanocortin receptor agonist PL-8331 is a potent therapeutic drug to suppress inflammation, prevent retinal degeneration, and preserve the normal anti-inflammatory activity of RPE.

## 1. Introduction

Melanocortin signaling regulates skin and hair pigmentation, metabolism, and the resolution of inflammation [1,2,3,4]. Within the immune-privileged ocular microenvironment, the activation of inflammation is suppressed, and the melanocortin signals play a central role [5,6]. The neuropeptide alpha-melanocyte stimulating hormone (α-MSH) is constitutively expressed within the healthy ocular microenvironment [7]. The neuropeptide α-MSH suppresses the induction of inflammation by immune cells through three of the five known melanocortin receptors (MCr). The thirteen-amino-acid-long neuropeptide binds to all melanocortin receptors except for MC2r, to which only adrenocorticotropic hormone binds. Through MC1r and MC3r, α-MSH suppresses pro-inflammatory activity while promoting anti-inflammatory activity in macrophages and other innate immune cells [8,9,10,11,12,13]. Through MC5r, α-MSH induces activation of regulatory activity in T cells and suppressor cell activity in myeloid cells [14,15,16,17]. Cells of the retina express MC1r and MC5r, and α-MSH can mediate anti-inflammatory activity while protecting the retina through these receptors in uveitic and diabetic eyes [18,19,20,21,22].

In both uveitis and diabetic retinopathy, there is a progressive loss of cells in the retina. The inflammation of uveitis and the ischemia/reperfusion caused by capillary occlusions and hyperglycemia in diabetes strain cell survival and initiates programmed cell death [23,24]. There is a great need for therapy that can suppress inflammation and cell death in the retina. Melanocortin-based therapy is a potentially beneficial therapy. A series of designed α-MSH-analogs have been studied as possible substitutes for α-MSH in melanocortin-based therapeutic approaches [22,25,26,27]. One of these α-MSH-analogs is Palatin Technologies PL-8331 [22]. The PL-8331 peptide is a pan-agonist that binds all the melanocortin receptors except MC2r and is more like α-MSH in regulating inflammation, including uveitis [22,26]. In addition, PL-8331 has 33-, 8-, 3-, and 21-fold greater functional activity than α-MSH on MC1r, MC3r, MC4r, and MC5r, respectively, and with greater in vivo stability [22]. Therefore, we assayed for the effects of augmenting the melanocortin signal with PL-8331 on the preservation of retinal cells and structure in two mouse models of retinal degeneration, experimental autoimmune uveoretinitis (EAU) and diabetic retinopathy (DR).

## 2. Results

### 2.1. Effects of PL-8331 Treatment on EAU

**Figure 1:**
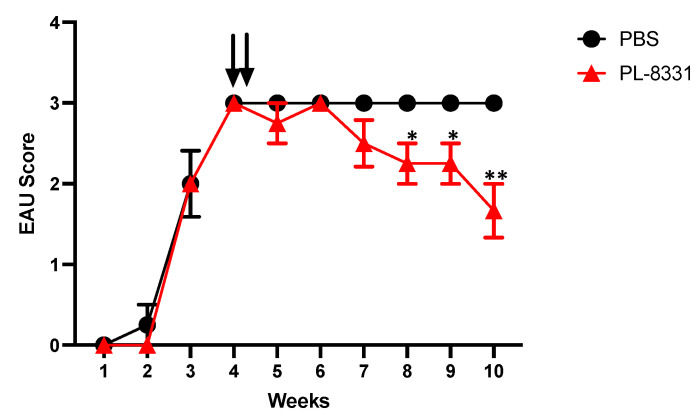
Effects of treating EAU mice with PL-8331. Mice were immunized to induce EAU, and the retinas were examined by microscopy and scored. The mice were treated with PL-8331 on week 4, as indicated by the arrows. Presented are the mean ± SEM of the EAU scores of each treated group over the weeks after immunization to induce EAU. Statistical differences of EAU clinical scores from PBS (vehicle)-treated EAU mice are indicated, * *p* ≤ 0.05, ** *p* ≤ 0.01; N = 4. All other values were not statistically different between groups.

**Figure 2:**
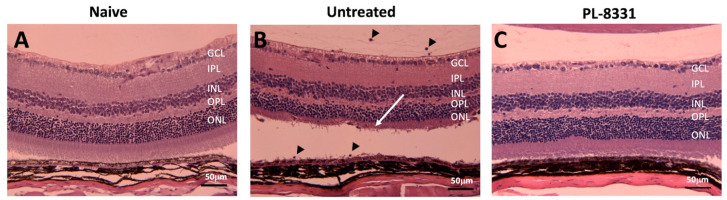
The effects of PL-8331 treatment on retinal histology in EAU mice. Photomicrographs of the histology of retinas from treated mice 10 weeks after immunization with IRBP and treatment. (**A**) The retinal structure of naive mice demonstrated normal thickness and lengthy photoreceptors. (**B**) The retinas of the EAU mice treated with PBS (vehicle) had noticeably shortened photoreceptor lengths with disruptions of the ONL (arrow). Also, there were infiltrating inflammatory cells (arrowheads). (**C**) The retinal structure in EAU mice treated with PL-8331 has a similar appearance as the retinas of naive mice. The separation of the retina from the RPE was an artifact of the fixation. Four sections of the eyes from the 4 mice in Figure 1 were examined. The scale bar is 50 μm in length. GCL—Ganglion Cells Layer, IPL—Inner Plexiform Layer, INL—Inner Nuclear Layer, OPL—Outer Plexiform Layer, ONL—Outer Nuclear Layer, Phr—Photoreceptors.

The mice were immunized to induce EAU, and the retinas were clinically scored by fundus microscopy for 10 weeks after immunization. On week 4, as the eyes entered the chronic phase of EAU, the mice received two consecutive intraperitoneal injections of PL-8331 at the molar equivalent of a therapeutic injection of *α*-MSH peptide [26] or vehicle (PBS). The PL-8331-treated EAU mice had significantly suppressed EAU clinical scores 6 weeks after treatment compared to vehicle-injected EAU mice (Figure 1). The eyes were collected to see if PL-8331 treatments affected retinal structures. The eyes were collected on week 10, along with the eyes of age-matched naive mice. They were sectioned and stained for histological analysis (Figure 2). The retinas of vehicle-injected EAU mice (Figure 2B) had increased infiltration of immune cells in the vitreous and visibly shortened photoreceptor lengths with disruption of the outer nuclear layer (ONL). In addition, the retinas showed inflammatory cells in the subretinal space near the disrupted ONL. In contrast, the retinas from the PL-8331-treated EAU mice (Figure 2C) retained almost normal retinal structures compared to retinas from naive mice (Figure 2A). Therefore, treating EAU with the *α*-MSH-analog PL-8331 suppresses the inflammation of uveitis and can preserve retinal structures.

### 2.2. Effects of PL-8331 Treatment on Diabetic Retinopathy

**Figure 3:**
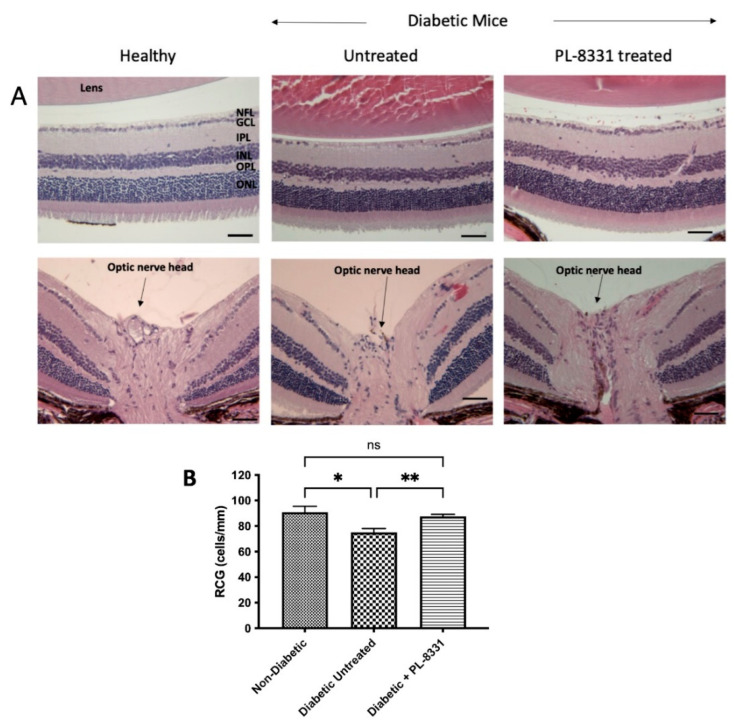
The effects of PL-8331 treatment on retinal cell survival in diabetic mice. The eyes of PL-8331-treated or untreated mice with diabetes for 16 weeks were collected, sectioned, hematoxylin and eosin stained, and retinal sections were examined. (**A**) The upper panels are cross-sections of the retina, and the lower panels are through the optic nerve head of the retina. There were more areas of RGC dropout in the GCL (arrow) of untreated diabetic mice compared to the retinas of non-diabetic mice and PL-8331-treated diabetic mice. Presented are representative sections from 10 sections per 10 eyes. NFL—Nerve Fiber Layer; GCL—Ganglion Cell Layer; IPL—Inner Plexiform Layer; INL—Inner Nuclear Layer OPL—Outer Plexiform Layer; ONL—Outer Nuclear Layer; Scale Bar: 50 µm in length. (**B**) From the hematoxylin-stained sections, the RGC nuclei per millimeter were counted from 10 retinal sections per 10 mouse eyes. There was a significant decrease in the number of RGC nuclei in the untreated diabetic retinas (* *p* ≤ 0.05). Treatment with PL-8331 significantly (** *p* ≤ 0.01) preserved the number of RGC nuclei in the diabetic mouse retinas.

Since there is a potential benefit of α-MSH-therapy to prevent diabetic retinopathy [18,20,21], we assayed for the possibility of PL-8331-therapy providing a similar benefit. We used a streptozotocin-induced diabetic retinopathy mouse model. After the last injection of streptozotocin, the diabetic mice were treated with intravitreal injections of PL-8331 every 4 weeks. At week 16, the eyes were collected and assayed for histology (Figure 3). The ganglion cell layer (GCL) in the diabetic eyes had gaps with no cells (arrow) in comparison to the retinas of non-diabetic mice and PL-8331-treated diabetic mice (Figure 3A Upper Panels). There was a small dent in the optic nerve head of the untreated retina of diabetic mice but not in the optic nerve head of non-diabetic and the PL-8331-treated diabetic mice suggesting preservation of optic nerve fibers (Figure 3A Lower Panels). No change was seen in retinal thickness between any of the groups of mice (Supplementary Materials Figure S1). However, there are observable regions of substantial RGC dropout in the diabetic mouse retinas (Figure 3A, white arrow). Such regions were not observed in the retinal sections of non-diabetic and PL-8331-treated diabetic mice. There was significant retention in the number of nuclei in the GCL of the PL-8331-treated diabetic mice (Figure 3B). The PL-8331 treatment of diabetic mice can potentially protect the retina from ganglion cell loss. This is like what is seen with α-MSH-treated diabetic mice in that retinal cells are protected [18,21].

**Figure 4:**
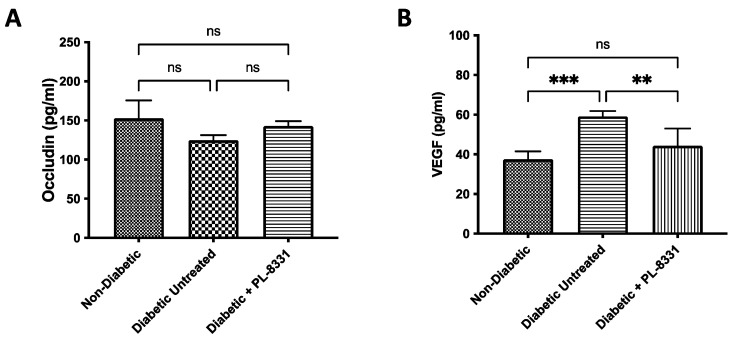
Effects of PL-8331 on the neuroretinal expression of Occludin and VEGF. The neuroretina was collected, homogenized, and assayed by ELISA for Occludin and VEGF. (**A**) The level of Occludin in the neuroretinal homogenates from diabetic mice was not significantly (ns) different from non-diabetic or PL-8331-treated diabetic mice. (**B**) The levels of VEGF expressed in neuroretinal homogenates from diabetic mice were significantly greater than those from the neuroretina of diabetic mice treated with PL-8133 (** *p* ≤ 0.01) and non-diabetic mice (*** *p* ≤ 0.005); N = 10. There was no statistical (ns) difference in neuroretinal VEGF levels between non-diabetic and PL-8331-treated diabetic mice.

In addition to the loss of cells, there are changes in intercellular connections and regulation of the microcirculation of diabetic retinas [18]. Homogenates of the neuroretina were assayed for the expression of the tight junction protein Occludin and the angiogenic factor vascular endothelial growth factor (VEGF) (Figure 4). While the Occludin levels are lower in diabetic retinas, they were not significantly different between the groups (Figure 4A). The levels of VEGF were significantly greater in the untreated diabetic mouse retinas (Figure 4B) than in the retinas of non-diabetic and PL-8331-treated diabetic mice. The suppression of enhanced VEGF levels is consistent with previous studies demonstrating that melanocortin signaling pathways regulate changes in retinal microvasculature [18,20]. 

One of the know effects of α-MSH-therapy in EAU is the restoration of RPE-mediated anti-inflammatory activity [6,19]. This is important for maintaining the normal anti-inflammatory microenvironment of the retina. Soluble factors produced by the RPE mediate this anti-inflammatory activity, which includes α-MSH. The RPE factors suppress the induction of proinflammatory activity while promoting anti-inflammatory activity by activated macrophages [19,28]. In addition, suppressing the inflammatory response in diabetes may prevent the development of diabetic retinopathy [29,30].

**Figure 5:**
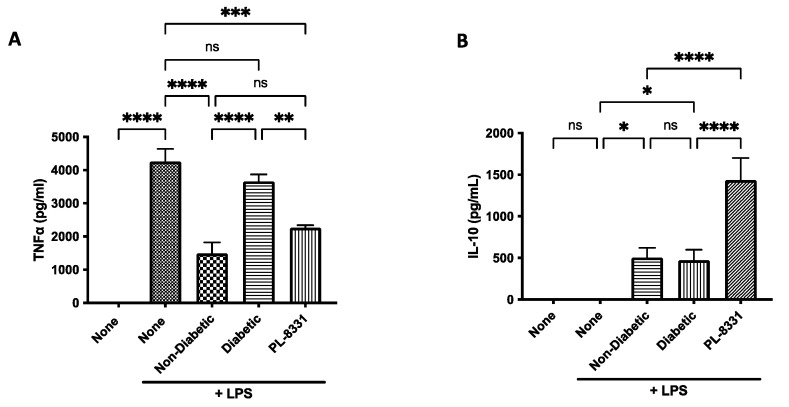
The effects of PL-8331-treatment of diabetes on RPE regulation of LPS-stimulated macrophages. The RPE eyecup conditioned media (CM) were collected from 24 h cultures of RPE eyecups of non-diabetic, diabetic, and PL-8331-treated diabetic mice. The macrophages were treated with the CM, stimulated with LPS, and 24 hrs later, the culture media was assayed for (**A**) TNFα and (**B**) IL-10 by ELISA. (**A**) The production of TNF-α from LPS-stimulated macrophages treated with RPE eyecup CM from non-diabetic mice was significantly suppressed, as well as the macrophages treated with RPE eyecup CM from PL-8331-treated diabetic mice. There were no statistical differences between the effects of the RPE eyecup CM in non-diabetic and PL-8331-treated diabetic mice. The RPE eyecup CM from diabetic mice did not suppress TNF-α production by the macrophages. (**B**) The production of IL-10 from LPS-stimulated macrophages treated with RPE eyecup CM from PL-8331-treated diabetic mice was significantly enhanced over the amount of IL-10 from macrophages treated with the CM of RPE eyecups from non-diabetic and diabetic mice. Statistical differences between the mean (pg/mL) ± SEM of the cultures were calculated (* *p* ≤ 0.05, ** *p* ≤ 0.01, *** *p* ≤ 0.005, **** *p* ≤ 0.001, ns = not significantly different; N = 10). An effect of treating diabetic mice with PL-8331 is the restoration or maintenance of RPE regulation of the macrophage response to proinflammatory stimuli.

To see whether RPE maintained its ability to suppress inflammation in the PL-8331-treated diabetic mice, the eyes were collected after treatment and dissected to generate RPE eyecups. The eyecups were cultured for 24 h, and the conditioned media (CM) was used to treat macrophages. The macrophages were then stimulated with LPS and assayed for TNF-α (a pro-inflammatory cytokine) and IL-10 (an anti-inflammatory cytokine). The CM from the RPE eyecups of diabetic mice did not suppress TNF-α production by LPS-stimulate macrophages compared to the suppression mediated by the CM from non-diabetic RPE eyecups (Figure 5A). The CM of RPE eyecups from PL-8331-treated diabetic mice significantly suppressed LPS-stimulated TNF-α production by the macrophages (Figure 5A). While there is significant production of IL-10 by the LPS-stimulated macrophages treated with the CM of RPE eyecups from non-diabetic and diabetic mice, the CM of RPE eyecups from PL-8331-treated diabetic mice had significantly enhanced macrophage production of IL-10 (Figure 5B). This demonstrated that the RPE in diabetic mice treated with PL-8331 retained their ability to mediate anti-inflammatory activity. This contrasts with RPE from untreated diabetic mice that permitted macrophage production of the proinflammatory cytokine TNF-α. Therefore, PL-8331 treatment of diabetic mice protects the retina while preserving RPE suppression of inflammation and possibly preventing changes in the retinal microvasculature.

## 3. Discussion

The melanocortins are a family of highly conserved endogenous peptides and receptors that influence various biological activities [3,31,32]. While initially characterized for inducing melanogenesis in frogs, the melanocortins regulate metabolism, sexual functionality, mood, and immunity. The prototypical melanocortin is α-MSH, constantly present in the eye and produced by the RPE [6,7]. The melanocortin receptors MC1r and MC5r are expressed in the eye and may be needed to maintain retinal structure and cell survival [18,19,21,33]. These same receptors are expressed on immune cells through which α-MSH suppresses the activation of macrophages and T cells that mediate inflammation [19,22,34]. In addition, α-MSH treatment may signal retinal cell survival or inhibit apoptosis signals [6,18,33,35,36]. It has been demonstrated that using α-MSH as a treatment protects the retina from degeneration caused by EAU, diabetic retinopathy, and ischemia/reperfusion [18,19,21,26,37,38,39,40]. While experimentally using α-MSH has demonstrated benefits, α-MSH is a highly unstable molecule [41]. The α-MSH-analog PL-8331 is highly stable and has as much as a 33-fold greater functional stimulation of the melanocortin receptors than α-MSH [22]. Also, like α-MSH, PL-8331 has no functional activity on MC2r, meaning no associated glucocorticoid response [42]. Our results showed that PL-8331 effectively suppressed retinal degeneration caused by inflammation and diabetes.

The retinal damage of EAU is the inflammatory response mediated by activated immune cells of an autoimmune-disease response [23]. Activating effector T cells with specificity to presented retinal antigens leads to the release of cytokines and chemokines that promote infiltration of other immune cells and the breakdown of the blood barrier that results in retinal degeneration. The suppression of EAU by PL-8331-treatment showed that by activating the melanocortin-signaling pathways, there was the suppression of inflammation and preservation of retinal structure similar to what others have seen using native α-MSH [16,19,43,44,45]. The literature describes the potential of stimulating the melanocortin signaling pathways to promote the survival of retinal cells [19,27,33,37,43,46,47,48]. The histology of the EAU retinas showed that PL-8331 treatment observably protects the photoreceptor layer. The PL-8331 therapy provided the combined benefit of suppressing inflammation with promoting retinal cell survival. This therapeutic approach of augmenting the melanocortin signal can minimize the damage of inflammation while potentially reinforcing or restoring the normal anti-inflammatory microenvironment of the eye while preserving vision.

In the diabetic model, hyperglycemia induces the expression of proinflammatory signals along with reactive oxidants in the retina [24]. In addition, there is retinal capillary damage and dropout. These can lead to localized areas of ischemia, edema, and angiogenesis. The possibility of targeting the MC1r and MC5r has been shown to reduce the retinopathy mediated by diabetes [18,40]. Our results demonstrated that PL-8331 therapy, a peptide that also targets MC1r and MC5r [22], effectively protected the eye from retinopathy associated with diabetes. The retinas of the diabetic mice treated with PL-8331 had preserved retinal structure with possible RGC survival and suppressed VEGF levels that would be expected to reduce the chance of developing diabetic retinopathy. It has been found that under diabetic conditions, α-MSH protects vascular endothelial cells from apoptosis and oxidative stress [40]. Also, there is the potential that α-MSH can modulate the release of VEGF from RPE under hyperglycemic conditions [18,20]. The treatment with PL-8331 preserved the normal anti-inflammatory activity of the RPE in diabetic mice. Diabetic patients with elevated pro-inflammatory cytokines in their serum have a greater risk of developing diabetic retinopathy [29,30]. In contrast, elevated serum IL-10 decreases the risk of developing diabetic retinopathy. The benefits we see with PL-8331-therapy in the diabetic eye could be associated with enhancing the normal anti-inflammatory microenvironment of the retina [5]. The results demonstrate the potential of PL-8331 treatment in diabetes to protect the eye from developing diabetic retinopathy.

In this study, we investigated the protective effect of PL-8331, an α-MSH analog, in treating two retinal disease models that cause retinal cell loss, retinal structural damage, and inflammation. The therapeutic use of PL-8331 provided the same effects as has been seen using α-MSH-peptide but with a comparatively more potent and stable analog [22]. While we used only one dose of PL-8331, it was equivalent to the molar concentration we previously used with α-MSH treatments. It is to be seen how long PL-8331 can last in the eye; however, since we injected the EAU mice twice and the diabetic eyes once every four weeks, its effects appear to last. In addition, the ocular injections of PL-8331 appear to be a safe route of administration as others have done with α-MSH [18,39,43]. Our results demonstrated the beneficial effects of PL-8331 on preserving the retina’s health under conditions that cause retinopathy. In addition, our findings further support evaluating the therapeutic potential of engaging endogenous melanocortin signaling pathways in ocular and other inflammatory diseases [22,25,42,49].

## 4. Materials and Methods

### 4.1. Animals

All mouse procedures described in this study were approved by the Boston University Institutional Animal Care and Use Committee (Protocol PROTO201800162) and adhered to the Association for Research in Vision and Ophthalmology (ARVO) Statement for the Use of Animals in Ophthalmic and Vision Research. The C57BL/6j mice were purchased from Jackson Laboratories (Bar Harbor, ME, USA) and housed in the Boston University Animal Science Center.

### 4.2. Experimental Autoimmune Uveoretinitis (EAU) Model

Mice were immunized for EAU, as previously described [19]. Briefly, the mice were injected with a 200 µL emulsion of complete Freund’s adjuvant (CFA) with 5 mg/mL desiccated M. tuberculosis (Difco Laboratories, Detroit, MI, USA) and 2 mg/mL IRBP peptide amino acids 1–20 (IRBP) (Genscript, Piscataway, NJ). This was immediately followed by an intraperitoneal injection of 0.3 µg pertussis toxin (Sigma-Aldrich, St. Louis, MO, USA) repeated 2 days later. Every 3–4 days, the retinas were examined using a slit lamp microscope with the cornea flattened and numbed with 0.5% proparacaine (Akorn, Lake Forest, IL, USA). The iris was dilated with 1% tropicamide (Akorn). As previously described, the clinical scores were on a 5-point scale based on the clinical signs of observable vessel dilatation, white focal lesions, and the extent of retinal vessel exudate, hemorrhage, and detachment [19]. The eyes were scored 0 for no inflammation; a score of 1 for only white focal lesions of the vessels; a score of 2 for linear vessel lesions over less than half of the retina; a score of 3 for linear vessels lesions over more than half of the retina; a score of 4 for severe chorioretinal exudates or retinal hemorrhages in addition to the vasculitis; and a score of 5 for subretinal hemorrhaging or retinal detachment. When all the mice reached a clinical score of 3 (4 to 5 weeks after immunization), the maximum score under our current animal facility conditions, the mice were treated with an intraperitoneal injection of PL-8331 (0.3 mg/kg/mouse) (Palatin Technologies Inc., Cranbury, NJ, USA) and again 2 days later. At 10 weeks after immunization, when the EAU of the PL8331-treated mice reached significantly sustained suppression of uveitis, the eyes were fixed with Davidson fixative for paraffin sectioning. The paraffin-embedded eyes were sectioned at 5 µm thickness. The sections were hematoxylin and eosin stained and imaged using an Olympus CX33 with the QCamera setup (Olympus).

### 4.3. Diabetic Retinopathy Model

The C57BL/6j mice at age 8 weeks were injected with a low-dose intraperitoneal injection of streptozotocin (40 mg/kg in 10 mM citrate buffer, pH 4.5) each day for 7 days. Streptozotocin was purchased from Sigma-Aldrich. Development of hyperglycemia was verified 1 week after the streptozotocin injection using a glucometer (Precision Xtra, Abbott Diabetes Care Inc., Alameda, CA, USA) (Supplemental Figure S2). The diabetic state was determined when the blood glucose was greater than 250 mg/dL. The streptozotocin-injection mice with lower glucose levels were excluded from the study. Blood glucose levels were checked every two weeks throughout the study to confirm the maintenance of the diabetic condition (Supplemental Figure S2). The diabetic mice were treated with one intravitreal injection of 1 µL of 3.3 µM PL-8331 or PBS-carrier (untreated control) on weeks 1, 4, 8, 12, and 16 after the onset of diabetes. After 16 weeks, one set of eyes was fixed in Davidson fixative for 2 days, then processed for paraffin sectioning and hematoxylin and eosin stained. The paraffin-embedded eyes were sectioned at 5 µm thickness. The sections were hematoxylin and eosin stained and imaged using an Olympus CX33 with the QCamera setup (Olympus). Another set of eyes was dissected in ice-cold PBS to make RPE eyecups. In addition, the neuroretina was homogenized on ice using RIPA buffer (Santa Cruz Biotechnology, Dallas, TX, USA) with protease inhibitors. The lysates were centrifuged, and the supernatant was assayed for protein concentration, VEGF (Quantikine ELISA kits, R&D Systems, Minneapolis, MN, USA), and Occludin (MyBioSource, San Diego, CA, USA) by ELISA.

### 4.4. RPE Eyecup Conditioned Media (CM)

The RPE eyecups were made as previously reported [19]. The enucleated eyes were dissected, making RPE eyecups consisting of the RPE monolayer with underlying choroid and sclera with the neuroretina removed. The RPE eyecups were placed into the wells of a 96-well round bottom tissue culture plate (Corning, Corning, NY, USA) containing 200 µL of serum-free media (SFM) (RPMI 1640 supplemented with 10 mM HEPES, 1 mM sodium pyruvate, and nonessential amino acids (Lonza), along with 0.2% ITS+1, 0.1% BSA, and 10 µg/mL gentamycin (Sigma-Aldrich). The cultures were incubated for 24 h at 37 °C in 5% CO_2_. The conditioned media (CM) was collected, centrifuged, and the supernatants were used as RPE eyecup-CM.

### 4.5. Macrophages and Treatment with RPE-Eyecup CM

The macrophages used in this study were the macrophage cell line RAW 264.7 (T1B-71, ATCC, Manassas, VA, USA). The RAW 264.7 cells were maintained in DMEM with 10% FBS (Lonza) and were passed three times after thawing before use. The cells were kept in culture for no more than 3 months and passed twice per week. The RAW 264.7 cells were seeded onto the wells of a 24-well culture plate at 3.8 × 10^5^ cells/mL and incubated for 1 h. The media was replaced with SFM and treated with the diabetic RPE eyecup-CM or naive RPE eyecup-CM for 30 min. Then the macrophages were stimulated with 1 µg/mL *E. coli* lipopolysaccharides (Sigma-Aldrich) and incubated in 5% CO_2_ at 37 °C for 24 h. The culture supernatants were collected for analysis of TNF-α and IL-10 using DuoSet ELISA kits (RnD Systems, Minneapolis, MN, USA).

### 4.6. Histology

The eyes were fixed in 4% paraformaldehyde in PBS for 48 h. After dehydration, the eyes were embedded in paraffin and sectioned at 5 µm thickness. The sections were hematoxylin and eosin stained and imaged using an Olympus CX33 with the QCamera setup (Olympus). The number of hematoxylin-stained nuclei was counted, and the retina length was measured using NIH ImageJ software v1.54d.

### 4.7. Statistical Analysis

The statistical analysis of the EAU clinical scores was a two-way ANOVA with non-parametric post-test analysis. Differences in cytokine levels and the number of counted nuclei in the RGC layer were assayed by ordinary one-way ANOVA with Dunnett’s post-analysis multiple comparisons. Significance was detected when the *p*-value was less than 0.05. Statistical calculations were done using PRISM 9 (GraphPad Software v9.5.1, San Diego, CA, USA), and the results were presented as the mean ± SEM for each experimental group of 4 to 10 unpaired eyes. The micrographs of the retinal sections are representative images that correspond to the mean of each experimental group.

## Data Availability

Not applicable.

## Supplementary Materials

The following supporting information can be downloaded at: https://www.mdpi.com/article/10.3390/ijms24086928/s1.

## References

[1] Perretti M., Leroy X., Bland E.J., Montero-Melendez T. (2015). Resolution Pharmacology: Opportunities for Therapeutic Innovation in Inflammation. Trends Pharm. Sci..

[2] Bohm M., Luger T.A., Tobin D.J., Garcia-Borron J.C. (2006). Melanocortin receptor ligands: New horizons for skin biology and clinical dermatology. J. Investig. Dermatol..

[3] Rees J.L. (2003). Genetics of hair and skin color. Annu. Rev. Genet..

[4] Fehm H.L., Born J., Peters A. (2004). Glucocorticoids and melanocortins in the regulation of body weight in humans. Horm. Metab. Res..

[5] Taylor A.W., Ng T.F. (2018). Negative regulators that mediate ocular immune privilege. J. Leukoc. Biol..

[6] Kawanaka N., Taylor A.W. (2011). Localized retinal neuropeptide regulation of macrophage and microglial cell functionality. J. Neuroimmunol..

[7] Taylor A.W., Streilein J.W., Cousins S.W. (1992). Identification of alpha-melanocyte stimulating hormone as a potential immunosuppressive factor in aqueous humor. Curr. Eye Res..

[8] Getting S.J., Lam C.W., Chen A.S., Grieco P., Perretti M. (2006). Melanocortin 3 receptors control crystal-induced inflammation. FASEB J..

[9] Lam C.W., Getting S.J. (2004). Melanocortin receptor type 3 as a potential target for anti-inflammatory therapy. Curr. Drug. Targets Inflamm. Allergy.

[10] Ignar D.M., Andrews J.L., Jansen M., Eilert M.M., Pink H.M., Lin P., Sherrill R.G., Szewczyk J.R., Conway J.G. (2003). Regulation of TNF-alpha secretion by a specific melanocortin-1 receptor peptide agonist. Peptides.

[11] Li D., Taylor A.W. (2008). Diminishment of alpha-MSH anti-inflammatory activity in MC1r siRNA-transfected RAW264.7 macrophages. J. Leukoc. Biol..

[12] Montero-Melendez T., Patel H.B., Seed M., Nielsen S., Jonassen T.E., Perretti M. (2011). The melanocortin agonist AP214 exerts anti-inflammatory and proresolving properties. Am. J. Pathol..

[13] Taherzadeh S., Sharma S., Chhajlani V., Gantz I., Rajora N., Demitri M.T., Kelly L., Zhao H., Ichiyama T., Catania A. (1999). alpha-MSH and its receptors in regulation of tumor necrosis factor-alpha production by human monocyte/macrophages. Am. J. Physiol..

[14] Lee D.J., Preble J., Lee S., Foster C.S., Taylor A.W. (2016). MC5r and A2Ar Deficiencies During Experimental Autoimmune Uveitis Identifies Distinct T cell Polarization Programs and a Biphasic Regulatory Response. Sci. Rep..

[15] Lee D.J., Taylor A.W. (2011). Following EAU recovery there is an associated MC5r-dependent APC induction of regulatory immunity in the spleen. Invest. Ophthalmol. Vis. Sci..

[16] Lee D.J., Taylor A.W. (2013). Both MC5r and A2Ar are required for protective regulatory immunity in the spleen of post-experimental autoimmune uveitis in mice. J. Immunol..

[17] Taylor A.W., Kitaichi N., Biros D. (2006). Melanocortin 5 receptor and ocular immunity. Cell. Mol. Biol. (Noisy-Le-Grand. Fr.).

[18] Rossi S., Maisto R., Gesualdo C., Trotta M.C., Ferraraccio F., Kaneva M.K., Getting S.J., Surace E., Testa F., Simonelli F. (2016). Activation of Melanocortin Receptors MC 1 and MC 5 Attenuates Retinal Damage in Experimental Diabetic Retinopathy. Mediat. Inflamm..

[19] Ng T.F., Manhapra A., Cluckey D., Choe Y., Vajram S., Taylor A.W. (2021). Melanocortin 5 Receptor Expression and Recovery of Ocular Immune Privilege after Uveitis. Ocul. Immunol. Inflamm..

[20] Maisto R., Oltra M., Vidal-Gil L., Martinez-Gil N., Sancho-Pelluz J., Filippo C.D., Rossi S., D’Amico M., Barcia J.M., Romero F.J. (2019). ARPE-19-derived VEGF-containing exosomes promote neovascularization in HUVEC: The role of the melanocortin receptor 5. Cell Cycle.

[21] Maisto R., Gesualdo C., Trotta M.C., Grieco P., Testa F., Simonelli F., Barcia J.M., D’Amico M., Di Filippo C., Rossi S. (2017). Melanocortin receptor agonists MCR1-5 protect photoreceptors from high-glucose damage and restore antioxidant enzymes in primary retinal cell culture. J. Cell. Mol. Med..

[22] Spana C., Taylor A.W., Yee D.G., Makhlina M., Yang W., Dodd J. (2018). Probing the Role of Melanocortin Type 1 Receptor Agonists in Diverse Immunological Diseases. Front. Pharm..

[23] Caspi R.R. (2010). A look at autoimmunity and inflammation in the eye. J. Clin. Invest..

[24] Wang W., Lo A.C.Y. (2018). Diabetic Retinopathy: Pathophysiology and Treatments. Int. J. Mol. Sci..

[25] Montero-Melendez T., Boesen T., Jonassen T.E.N. (2022). Translational advances of melanocortin drugs: Integrating biology, chemistry and genetics. Semin. Immunol..

[26] Ng T.F., Dawit K., Taylor A.W. (2022). Melanocortin receptor agonists suppress experimental autoimmune uveitis. Exp. Eye Res..

[27] Gesualdo C., Balta C., Platania C.B.M., Trotta M.C., Herman H., Gharbia S., Rosu M., Petrillo F., Giunta S., Della Corte A. (2021). Fingolimod and Diabetic Retinopathy: A Drug Repurposing Study. Front. Pharm..

[28] Lau C.H., Taylor A.W. (2009). The immune privileged retina mediates an alternative activation of J774A.1 cells. Ocul. Immunol. Inflamm..

[29] Lee J.H., Lee W., Kwon O.H., Kim J.H., Kwon O.W., Kim K.H., Lim J.B. (2008). Cytokine profile of peripheral blood in type 2 diabetes mellitus patients with diabetic retinopathy. Ann. Clin. Lab. Sci..

[30] Mysliwiec M., Zorena K., Balcerska A., Mysliwska J., Lipowski P., Raczynska K. (2006). The activity of N-acetyl-beta-D-glucosaminidase and tumor necrosis factor-alpha at early stage of diabetic retinopathy development in type 1 diabetes mellitus children. Clin. Biochem..

[31] Voisey J., Carroll L., van Daal A. (2003). Melanocortins and their receptors and antagonists. Curr. Drug. Targets.

[32] Hadley M.E., Dorr R.T. (2006). Melanocortin peptide therapeutics: Historical milestones, clinical studies and commercialization. Peptides.

[33] Cheng L.B., Cheng L., Bi H.E., Zhang Z.Q., Yao J., Zhou X.Z., Jiang Q. (2014). Alpha-melanocyte stimulating hormone protects retinal pigment epithelium cells from oxidative stress through activation of melanocortin 1 receptor-Akt-mTOR signaling. Biochem. Biophys. Res. Commun..

[34] Taylor A.W., Lee D.J. (2011). The alpha-melanocyte stimulating hormone induces conversion of effector T cells into treg cells. J. Transpl..

[35] Taylor A.W. (2013). Alpha-melanocyte stimulating hormone (alpha-MSH) is a post-caspase suppressor of apoptosis in RAW 264.7 macrophages. PLoS ONE.

[36] Vecsernyes M., Juhasz B., Der P., Kocsan R., Feher P., Bacskay I., Kovacs P., Tosaki A. (2003). The administration of alpha-melanocyte-stimulating hormone protects the ischemic/reperfused myocardium. Eur. J. Pharm..

[37] Goit R.K., Taylor A.W., Lo A.C.Y. (2022). Anti-inflammatory alpha-Melanocyte-Stimulating Hormone Protects Retina After Ischemia/Reperfusion Injury in Type I Diabetes. Front. Neurosci..

[38] Edling A.E., Gomes D., Weeden T., Dzuris J., Stefano J., Pan C., Williams J., Kaplan J., Perricone M.A. (2011). Immunosuppressive activity of a novel peptide analog of alpha-melanocyte stimulating hormone (alpha-MSH) in experimental autoimmune uveitis. J. Neuroimmunol..

[39] Cai S., Yang Q., Hou M., Han Q., Zhang H., Wang J., Qi C., Bo Q., Ru Y., Yang W. (2018). Alpha-Melanocyte-Stimulating Hormone Protects Early Diabetic Retina from Blood-Retinal Barrier Breakdown and Vascular Leakage via MC4R. Cell. Physiol. Biochem..

[40] Zhang L., Dong L., Liu X., Jiang Y., Zhang L., Zhang X., Li X., Zhang Y. (2014). alpha-Melanocyte-stimulating hormone protects retinal vascular endothelial cells from oxidative stress and apoptosis in a rat model of diabetes. PLoS ONE.

[41] Catania A., Gatti S., Colombo G., Lipton J.M. (2004). Targeting melanocortin receptors as a novel strategy to control inflammation. Pharm. Rev..

[42] Clemson C.M., Yost J., Taylor A.W. (2017). The Role of Alpha-MSH as a Modulator of Ocular Immunobiology Exemplifies Mechanistic Differences between Melanocortins and Steroids. Ocul. Immunol. Inflamm..

[43] Lee D.J., Biros D.J., Taylor A.W. (2009). Injection of an alpha-melanocyte stimulating hormone expression plasmid is effective in suppressing experimental autoimmune uveitis. Int. Immunopharmacol..

[44] Nishida T., Miyata S., Itoh Y., Mizuki N., Ohgami K., Shiratori K., Ilieva I.B., Ohno S., Taylor A.W. (2004). Anti-inflammatory effects of alpha-melanocyte-stimulating hormone against rat endotoxin-induced uveitis and the time course of inflammatory agents in aqueous humor. Int. Immunopharmacol..

[45] Shiratori K., Ohgami K., Ilieva I.B., Koyama Y., Yoshida K., Ohno S. (2004). Inhibition of endotoxin-induced uveitis and potentiation of cyclooxygenase-2 protein expression by alpha-melanocyte-stimulating hormone. Invest. Ophthalmol. Vis. Sci..

[46] Varga B., Gesztelyi R., Bombicz M., Haines D., Szabo A.M., Kemeny-Beke A., Antal M., Vecsernyes M., Juhasz B., Tosaki A. (2013). Protective effect of alpha-melanocyte-stimulating hormone (alpha-MSH) on the recovery of ischemia/reperfusion (I/R)-induced retinal damage in a rat model. J. Mol. Neurosci..

[47] Naveh N. (2003). Melanocortins applied intravitreally delay retinal dystrophy in Royal College of Surgeons rats. Graefes Arch. Clin. Exp. Ophthalmol..

[48] O’Steen W.K., Kastin A.J. (1980). Relationship of melanocyte-stimulating hormone to photoreceptor damage. Peptides.

[49] Nelson W.W., Lima A.F., Kranyak J., Opong-Owusu B., Ciepielewska G., Gallagher J.R., Heap K., Carroll S. (2019). Retrospective Medical Record Review to Describe Use of Repository Corticotropin Injection Among Patients with Uveitis in the United States. J. Ocul. Pharm..

