# Supplementary material for: Stimulating the Melanocortin System in Uveitis and Diabetes Preserves the Structure and Anti-Inflammatory Activity of the Retina
Source: Int J Mol Sci. 2023 Apr 8;24(8):6928. doi: 10.3390/ijms24086928 (PMC10138492; doi:10.3390/ijms24086928)
Supplement: Supplementary file 1 [file ijms-24-06928-s001.zip › ijms-2240017-supplementary.pdf]

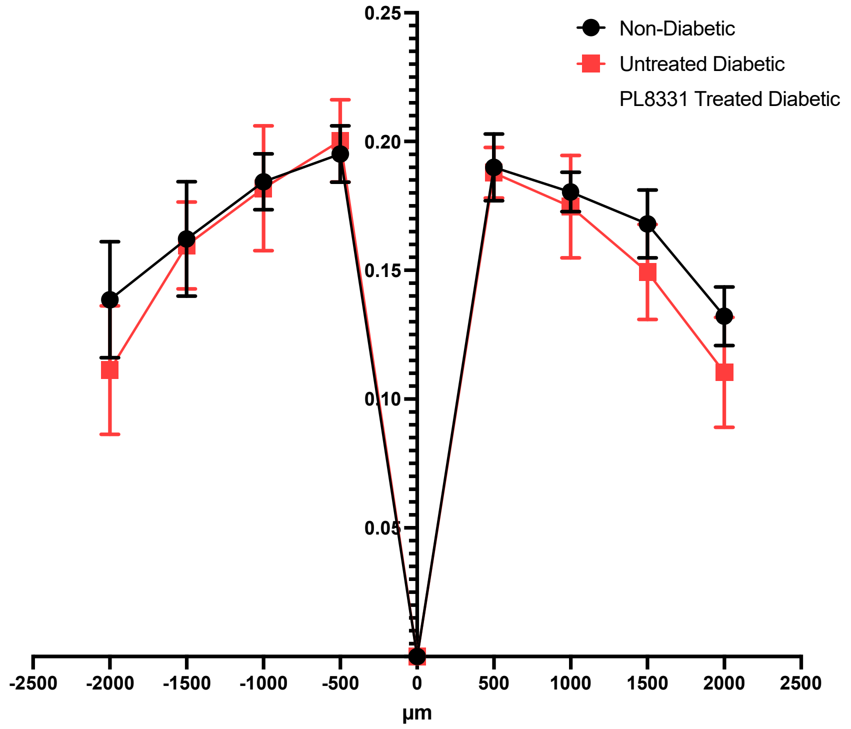

Supplemental Figure S1. The effects of PL-8331 treatment on whole retinal thickness in diabetic mice. The eyes of PL-8331-treated or untreated mice with diabetes for 16 weeks were collected, sectioned, hematoxylin and eosin stained, and whole retinal thickness was measured. Presented are the mean mm ± SD of the retinas from 8 mice per group at specific intervals along the retina from the optic nerve (0 μm). Compared to non-diabetic mice, there was no statistically significant difference between the groups of treated and untreated diabetic mice.

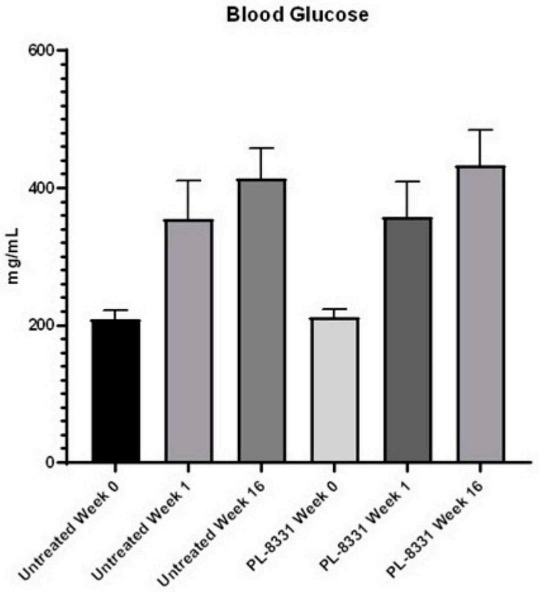

Supplemental Figure S2: Blood glucose level after streptozotocin injection: Blood glucose was measured before streptozotocin injection and then 1 week and 16 weeks later. Blood glucose levels in all animals at week 1 and week 16 were significantly higher than in week 0 before the streptozotocin injection.
